# Higher-order genome architecture of spermatids guides chromatin assembly in mature sperm

**DOI:** 10.21203/rs.3.rs-10308188/v1

**Published:** 2026-07-24

**Authors:** Mashiat Rabbani, Zachary Apell, Timothy J. Parnell, Lindsay Moritz, Sion Kim, Sowmya Srinivasan, Ritvija Agrawal, Alexander Vargo, Peter Orchard, Wenxin Xie, Lydia Freddolino, Alan P Boyle, Jun Z. Li, Bluma J. Lesch, Bradley Cairns, Minji Kim, Thomas E. Wilson, Saher Sue Hammoud

**Affiliations:** 1. Department of Human Genetics, University of Michigan, Ann Arbor, MI, 48109; 2. Department of Biostatistics, University of Michigan, Ann Arbor, MI, 48109; 3. Gilbert S. Omenn Department of Computational Medicine and Bioinformatics, University of Michigan, Ann Arbor, MI 48109; 4. Huntsman Cancer Institute, University of Utah School of Medicine, Salt Lake City, UT 84112; 5. Department of Electrical and Computer Engineering, University of Michigan, Ann Arbor, MI 48109; 6. Department of Biological Chemistry, University of Michigan, Ann Arbor, MI, 48109; 7. Department of Molecular Genetics and Genome Sciences, University of Oklahoma Health Science Center, College of Medicine, 73019; 8. Department of Genetics, Yale School of Medicine, New Haven, CT 06510; 9. Department of Obstetrics and Gynecology, University of Michigan, Ann Arbor, MI, 48109; 10. Department of Urology, University of Michigan, Ann Arbor, MI, 48109; 11. Department of Pathology, University of Michigan, Ann Arbor, MI, 48109

## Abstract

Differential packaging of vertebrate sperm and egg genomes is evolutionarily conserved for hundreds of millions of years. Unlike oocytes, post-meiotic spermatid nuclei are thought to uniformly replace histones with transition proteins (TNPs) and, ultimately, with protamines, the small non-histone structural proteins. Tracking of endogenously tagged protamines overturns the classical model of paternal chromatin remodeling, revealing a direct histone-to-protamine-1 transition that occurs independently of both TNPs and protamine-2. Stage-resolved chromatin profiling across 26 spermatid datasets reveals that chromatin compaction is a programmed process that requires H4-hyperacetylation but is not instructed by it. Rather, chromatin remodeling is guided by the pre-meiotic 3D-architecture of the spermatid nucleus: i.e., compaction of the A-compartment precedes B-compartment. This compartment-encoded template for sperm nuclear remodeling suggests that male-specific protamine patterns can transmit pre-meiotic structural information to the zygote and may impact embryonic development.

## Introduction

In sexually reproducing species, the sperm cell genome undergoes one of the most remarkable chromatin transformations in biology: the transition from a nucleosome-based to a protamine-based chromatin landscape. This process relies on a series of testis-specific basic proteins to achieve the tight chromatin compaction required for sperm function, while preserving the sperm genome’s ability to rapidly and faithfully reactivate after fertilization.^1,2^ The prevailing 'histone-to-protamine' exchange model depicts this chromatin transformation as a series of stepwise remodeling events in which canonical histones are first replaced by histone variants^3–6^, which overlap with transition proteins (TNP1 and TNP2), before being ultimately replaced by protamines (PRM1 and PRM2).^7–11^ This remodeling cascade implicitly assumes a linear, hierarchical exchange of proteins, in which each intermediate is a prerequisite for the next.

However, accumulating genetic evidence challenges this strict sequence of remodeling events. For example, single *Tnp1* or *Tnp2* knockouts in mice result in sub-fertility^8,10,12^, while double knockouts are male sterile^13^, indicating partial redundancy and cooperative roles in chromatin remodeling.^6, 7^ Notably, PRM1 incorporation occurs normally even in the complete absence of both transition proteins, whereas PRM2 incorporation is compromised, demonstrating that TNPs are required only for PRM2 deposition.^4,6,10^ Likewise, PRM1 deposition is unaffected in *Prm2*-deficient testes, establishing that PRM1 and PRM2 incorporation are mechanistically uncoupled.^8^ Together, these findings point to a modular chromatin-remodeling process, yet how this nuclear repackaging is regulated - and whether it follows a deterministic or stochastic logic - remains unknown.

Despite histone-to-protamine exchange being essential for sperm function, the exchange process is incomplete, with a small subset of histones retained^14–16^ at developmental gene promoters and at certain repetitive regions of the genome.^17–19^ These retained nucleosomes have been implicated in paternal epigenetic inheritance^14–17,20–22^ and are often presumed to be the only information carriers within the sperm nucleus. This view has prevailed because protamines are presumed to bind DNA uniformly and without sequence specificity. Yet this assumption has not been tested in vivo because of longstanding technical limitations. As a result, whether protamines transmit any information, locus- or compartment-level, to the zygote, or whether they function purely as structural condensation proteins with random genomic distribution, remains an open question.

Here, our findings challenge the established sequence of histone-to-protamine exchange events and the long-standing assumption that PRM1 and 2 are deposited uniformly throughout the paternal genome. By tagging the endogenous *Prm1* and *Prm2* loci with V5 and/or HA epitopes, we demonstrate that PRM1 and PRM2 incorporation is temporally uncoupled, with PRM1 undergoing a direct histone-to-PRM1 transition, whereas PRM2 incorporation occurs only after the appearance of transition proteins. This temporal uncoupling raised the possibility that PRM1 and PRM2 are not uniformly distributed across the genome. To test this, we applied stage-resolved ATAC-seq and CUT&Tag to capture successive waves of chromatin compaction and compositional change, as proxies for PRM1- and PRM2-driven accessibility changes. We hypothesized that if accessibility varies along the genome and across stages this can indicate that protamine exchange is a regionally partitioned process that likely encodes heterogeneity into the sperm chromatin landscape. Our dense, stage-resolved mapping of chromatin accessibility, nucleosome occupancy, and histone modifications indeed confirms a continuous, highly ordered, and progressive change in accessibility across spermiogenesis. This loss of chromatin accessibility coincides with the loss of H2B and H4, indicating that chromatin compaction is coupled to histone eviction. To examine whether H4 hyperacetylation regulates these stage-specific changes, we performed H4ac CUT&Tag across stages and show that H4 acetylation marks early, intermediate, and late remodeling loci- suggesting it accompanies but does not direct the remodeling process. Instead, integrating accessibility dynamics with 3D genome architecture in round spermatids showed that differential accessibility loss across stages is correlated with higher-order genome organization, with euchromatic A-compartment regions compacting earlier than heterochromatic B-compartment regions. Together, these results demonstrate that the temporal uncoupling of PRM1 and PRM2 coincides with a programmed, compartment-encoded remodeling process, suggesting that protamine 1 and −2 incorporation encodes compartment-level architectural information within the mature sperm nucleus.

## Results

### A revised temporal sequence of histone-to-protamine exchange.

**Figure 1:**
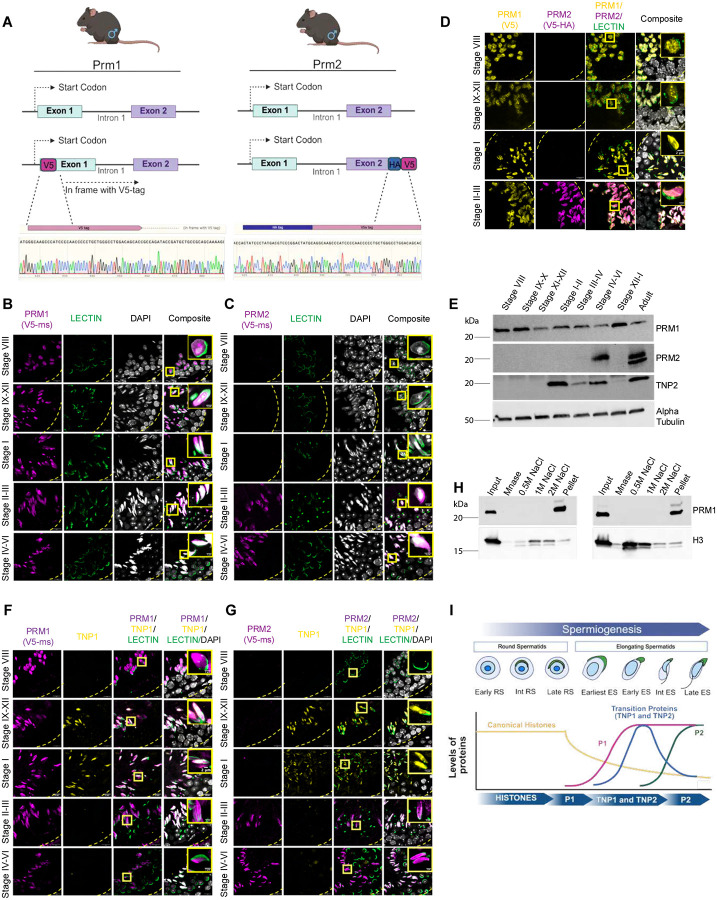
PRM1 undergoes direct histone exchange independently of and prior to PRM2 incorporation during spermiogenesis. **(A)** Schematic of the *Prm1-V5* and *Prm2-HA-V5* knock-in mouse models, with Sanger sequencing confirming in-frame insertion of the V5 tag at the N-terminus of PRM1 and dual HA-V5 tags at the C-terminus of PRM2. **(B)** Immunofluorescence of seminiferous tubule cross-sections from *Prm1-V5* heterozygous mice stained with anti-V5. Roman numerals denote the tubule stage, ordered here according to the sequential progression of histone-to-protamine exchange during spermiogenesis. PRM1 (anti-V5; Magenta); Lectin (green); DAPI (grey). Scale bar: 10 μm; inset: 2 μm. **(C)** As in (B), we co-stained testis cross-sections from *Prm2-V5* heterozygous mice with PRM2 (anti-V5; Magenta), Lectin (green), and DAPI (grey). Scale bar: 10μM, Inset Scale bar 2 μm. **(D)** Co-immunostaining of testes cross-sections spanning stages of seminiferous tubule, stained with anti-V5 and anti-HA in compound heterozygous *Prm1*^V5/+^; *Prm2*^V5-HA/+^ mice. Scale bar: 10μm, Inset Scale bar 2 μm. PRM1 (anti-V5; yellow); PRM2 (anti-HA; magenta), Lectin (green); DAPI (grey). **(E)** Immunoblots of PRM1, PRM2, and TNP2 from whole testes of WIN 18,446-synchronized mice collected at various days post-RA injection corresponding to seminiferous tubule stages VIII, IX–X, XI–XII, I–II, III–IV, V–VI, and IX–X. The adult testis serves as a positive control. **(F)** Co-staining for PRM1 and TNP1. Scale bar: 10 μM, Inset Scale bar 2 μm. PRM1 (magenta); TNP1 (yellow); Lectin (green); DAPI (grey). **(G)** Co-staining for PRM2 and TNP1. Scale bar: 10 μM, Inset Scale bar 2 μm. PRM2 (magenta); TNP1 (yellow); Lectin (green); DAPI (grey). **(H)** Western blot of nuclear lysates from WIN 18,446-synchronized testes isolated at stages IX and X post-RA injection. Chromatin was solubilized by MNase digestion, followed by sequential salt extractions (0.5–2 M NaCl), and immunoblotted for PRM1 and H3 to distinguish chromatin-bound from soluble protamine fractions. **(I)** Schematic for the revised sequence of PRM1 and PRM2 nuclear incorporation during histone-to-protamine exchange, as supported by this figure.

The precise temporal relationships among histones, transition proteins, and individual protamines in vivo have remained difficult to resolve due to limitations in available reagents and antibodies. To directly define the timing of protamine incorporation during spermiogenesis, and to monitor protamine deposition relative to other nuclear proteins under physiological conditions, we therefore used CRISPR-Cas9-mediated genome editing to introduce V5 or V5–HA epitope tags in frame into mouse *Prm1* and *Prm2* alleles, respectively (Figure 1A; note other tested tags led to infertility). Importantly, both tagged *Prm1* and *Prm2* alleles supported normal spermatogenesis, as evidenced by the normal testis histology, sperm counts, motility, and fertility relative to wild-type controls (Supplementary Figure 1A, B).

We first analyzed the *Prm1*-V5 and *Prm2*-V5–HA alleles independently to establish their individual timing of nuclear enrichment. Since spermatogonial stem cell differentiation is an asynchronous, highly regulated process, any given tubule cross-section contains germ cells at multiple stages of differentiation. These recurring cellular associations define the 12 stages of the seminiferous epithelium cycle^23,24^. Within these stages, spermatids mature through 16 morphologically distinct steps. Notably, histone-to-protamine exchange initiates at stage VIII (RS step 8) and proceeds sequentially through stages IX-XII (steps 9–12), I–III (steps 13–14), and IV-VII (steps 15–16). Using either anti-V5 for PRM1 or anti-V5/HA to track PRM2, we observed that PRM1 accumulates in spermatid nuclei at stage VIII and persists through subsequent stages (Figure 1B). In contrast, PRM2 nuclear enrichment was not detected until late stages of spermatid maturation (Stages II-VI; Figure 1C). These observations reveal a previously underappreciated temporal uncoupling between the two protamine proteins (Figure 1B, C). To determine whether this uncoupling reflects bona fide differences in protamine dynamics within the same cellular context - rather than variability across animals or sections - we next examined the relative incorporation of PRM1 and PRM2 within individual cells within a seminiferous tubule. Co-staining of testis sections from *Prm1*-V5; *Prm2*-V5-HA mice with antibodies against V5 and HA revealed the same reproducible temporal pattern observed in the single-allele analyses (Figure 1D). This result was independently confirmed by co-staining *Prm2*-V5–HA testes with an antibody against endogenous PRM1 (Hup1N) (Supplementary Fig. 1C). To further exclude that the observed uncoupling is due to differential nuclear accessibility of the antibodies to DNA-bound PRM1 vs. PRM2, we synchronized spermatid development by modulating retinoic acid signaling.^25^ This approach produces testes in which entire seminiferous tubules are restricted to one to two consecutive stages of the seminiferous cycle (e.g., VII- VIII, IX-X, XI-XII, etc.). Using testis samples containing spermatids at different stages, we performed immunoblots to monitor the dynamics of PRM1 and PRM2. Consistent with the immunofluorescence data, PRM1 protein was detected earlier than PRM2 across synchronized developmental windows (Figure 1E). Together, these complementary approaches establish that PRM1 and PRM2 translation and nuclear localization are temporally uncoupled and differentially regulated.

We next examined the temporal relationship between protamine incorporation and transition protein localization during spermiogenesis. Co-staining of Prm1^v5/+^ testis sections with anti-V5 and either TNP1 (Figure 1F) or TNP2 (Supplementary Figure 1D) revealed that PRM1 accumulates in spermatid nuclei beginning at stage VIII, before detectable nuclear localization of either transition protein, which first appears at stages IX-XII (Figure 1F). This temporal ordering was independently confirmed by immunoblots from the synchronized testes preparations from above, which revealed that PRM1 protein accumulation indeed precedes the appearance of TNPs (Figure 1E). In contrast, co-staining of PRM2^v5/+^ mouse testis sections with anti-V5 and either TNP1 (Figure 1G) or TNP2 (Supplementary Figure 1E) demonstrated that transition proteins precede PRM2 nuclear localization, consistent with the conventional model of transition protein-PRM2 incorporation order.

Although these data show that PRM1 precedes transition proteins, the PRM1 nuclear localization alone does not equate to chromatin association. To determine whether PRM1 in early spermatid is stably incorporated into chromatin or is in the nucleoplasm awaiting transition protein-mediated remodeling, we synchronized spermatogenesis in wild-type mice^25,26^, collected testis enriched for stages VIII or IX of the seminiferous tubule cycle, and subjected single-cell suspensions from these stages to sequential salt fractionation^27^ (Figure 1H). In stage VIII-enriched chromatin, histone H3 was progressively released with increasing salt concentration as expected, whereas PRM1 remained predominantly in the insoluble chromatin pellet even at high ionic strength, indicating stable chromatin association. Notably, the identification of PRM1 in the insoluble fraction is unlikely to be an artifact of aggregation due to disulfide bond formation, as disulfides only form during epididymal maturation^28–30^. This salt-resistant association persisted in stage IX-enriched chromatin, despite increased histone lability at this stage, consistent with the incorporation of testis-specific histone variants^5,6,31^ known to destabilize nucleosomes^32,33^. Together, these results demonstrate that PRM1 is stably chromatin-bound prior to, and independent of, transition protein nuclear accumulation. Consistent with a direct histone-to-PRM1 transition, co-staining of *Prm1*^V5/+^ testes for PRM1, histone H3, or acetylated Histone H4 (H4ac) revealed substantial temporal overlap between PRM1 and Histone H3 or PRM1 and H4ac in stage VIII spermatids (Supplementary Figure 1F,G), but in stage IX-XII the PRM1-high regions lose histone H3 or acetylated histone H4 (H4ac) (Supplementary Figure 1F,G), suggesting that PRM1 deposition coincides with histone H3 or H4ac loss (see CUT&Tag data below).

Together, these data establish a direct histone-to-PRM1 transition in which PRM1 is stably incorporated into chromatin prior to the accumulation of transition proteins and independently of PRM2. In contrast, PRM2 incorporation follows transition proteins, as previously described (Figure 1I). We therefore propose a revised sequence for the histone-to-protamine exchange: Histones → PRM1 → TNP1/2 → PRM2 (Figure 1I). This revised sequence of chromatin remodeling is strongly supported by earlier genetic data showing that PRM1 nuclear incorporation is preserved in *Tnp1/Tnp2* double-knockout testes, despite the complete absence of transition proteins, whereas PRM2 incorporation is compromised or lost in some spermatids^13^, indicating that PRM1 and PRM2 likely incorporate into chromatin through distinct mechanisms. More importantly, this direct histone-to-PRM1 exchange is not an idiosyncrasy of mouse spermiogenesis but has been reported in birds and certain fish, suggesting this is a likely conserved feature of vertebrate spermiogenesis^34,35^.

### Defining stage-specific architectural changes in sperm chromatin during spermiogenesis.

The temporal uncoupling of PRM1 and PRM2 incorporation raises the possibility that protamine deposition follows a staged, ordered compaction program rather than a single, uniform compaction event. A definitive test of this model would require mapping PRM1- and PRM2-bound chromatin at high resolution in sperm. However, the extreme compaction of protamine-bound DNA and the lack of accessible linker regions between protamine molecules in sperm make direct nuclease- or transposase-based foot-printing approaches technically impossible^36^. We therefore adopted an indirect but powerful strategy, using ATAC-seq^37,38^ to monitor chromatin accessibility dynamics across spermatid maturation.

**Figure 2:**
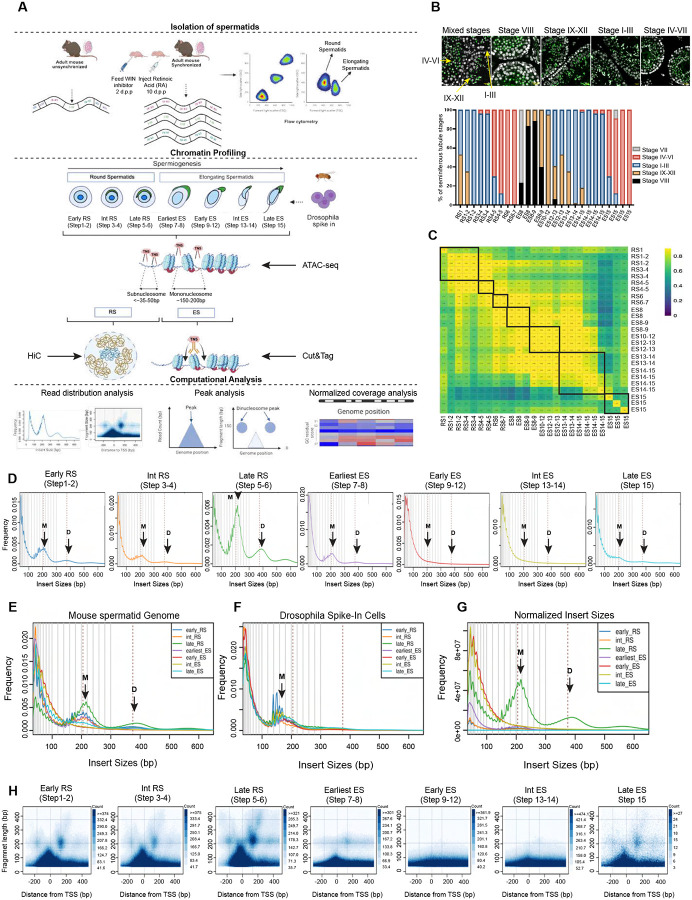
Stage-resolved chromatin profiling reveals progressive and programmatic loss of nucleosome footprint during spermiogenesis. **(A)** Schematic of the experimental design and computational framework for stage-specific chromatin profiling across spermiogenesis. Mouse spermatid populations were isolated from specific stages of the seminiferous tubule cycle by flow cytometry from unsynchronized and WIN 18,446/RA-treated testis and subjected to ATAC-seq, CUT&Tag, and Hi-C. Computational analyses included read distribution analysis, base-level peak calling, and GC-normalized bin-level coverage analysis. **(B)** Representative immunofluorescence images of seminiferous tubule cross-sections from unsynchronized and synchronized mice (top). Bar plot (bottom) shows the proportion of tubules at each seminiferous tubule stage across collected samples, confirming stage enrichment by synchronization. **(C)** Spearman correlation heatmap of all ATAC-seq samples. Biological replicates cluster tightly and samples separate by developmental stage. **(D)** ATAC-seq fragment length, i.e., insert size frequency distributions aggregated across the seven collapsed datasets of spermiogenesis. Solid vertical lines are size boundaries used during data analysis (see Methods). Arrows at 205 and 375 bp label the centers of the mononucleosome (M) and dinucleosome (D) fragment peaks in each stage. **(E)** Overlaid ATAC-seq fragment length distributions from early round to elongating spermatid populations. Arrows at 205 and 375 bp label the centers of the mononucleosome (M) and dinucleosome (D) fragment peaks. **(F)** Overlaid ATAC-seq fragment length distributions from *Drosophila* spike-in reads across all seven spermatid stages, for comparison to (E). Arrow at 165bp labels the center of the mononucleosome (M) fragment peak. **(G)** Overlaid fragment length distributions from (D) after normalizing to the corresponding *Drosophila* spike-in, to reveal changes in absolute chromatin accessibility between stages. Arrows at 205 and 375 bp label the centers of the mononucleosome (M) and dinucleosome (D) fragment peaks. **(H)** V-plots of ATAC-seq fragment lengths across all seven stages as a function of distance from the TSS of actively transcribed genes based on PRO-seq (Kaye et al., 2024). The characteristic promoter nucleosome pattern in round spermatids is progressively lost in elongating spermatid stages, reflecting stage-dependent disruption of promoter-proximal nucleosome positioning.

To collect spermatids at specific stages of development, we combined spermatogenesis synchronization^25^ with fluorescence-activated cell sorting^39^ (Figure 2A) to enrich for a homogeneous pool of spermatids isolated from any given stage (Figure 2B; Supplementary Table 1; **see Methods**). Importantly, because elongating spermatids are largely transcriptionally quiescent, the stage-specific changes in accessibility primarily reflect structural changes in chromatin rather than transcriptional activity.^40,41^ Therefore, the accessibility changes we observe can serve as a sensitive proxy for distinct compaction states dominated by histones, PRM1, or PRM1 plus PRM2 (Figure 2A).

To map chromatin accessibility dynamics over time, we densely sampled spermatid maturation, generating 26 ATAC-seq datasets spanning the 16 steps of spermatid maturation (Figure 2B; Supplementary Table 1). Each sample contained approximately 220,000 mouse cells supplemented with a 10% *Drosophila* spike-in to normalize across samples and to control for transposase activity and library complexity (Figure 2A). To assess reproducibility and global chromatin similarity across spermatid populations, we computed pairwise Spearman correlations of normalized signals across 10kb bins for all 26 datasets (Figure 2C). The resulting clustered heatmap revealed high concordance between biological replicates, which clustered tightly along the diagonal, confirming the robustness of our cell synchronization and sorting. Notably, correlations decreased progressively between non-adjacent stages, reflecting the continuous remodeling of the chromatin landscape during spermiogenesis (Figure 2C). Based on the correlation structure and confirmed biological sample stages (Figure 2B;2C; Supplemental Table 1), we binned samples into seven discrete windows of spermatid maturation (Figure 2A;2B; Supplementary Table 1) spanning early round spermatids (Early RS) through late elongating spermatids (Late ES; isolated from stages I-XII; Steps 1–15). These windows allow us to capture relevant developmental transitions while maintaining the granularity of the spermatid maturation trajectory. The latest elongating spermatid samples we could generate sufficient-quality ATAC-seq libraries without resorting to harsh chemical treatments, such as DTT, to increase chromatin accessibility are Step 15 spermatids isolated from Stage IV of the seminiferous tubule cycle.

By leveraging intrinsic ATAC-seq features, such as insert size distributions at specific genomic loci, such as transcriptional start sites (TSSs), or across the genome, the data provide a global, model-independent readout of the changing spermatid chromatin architecture. Early round spermatids displayed canonical nucleosome footprints, with prominent peaks at approximately 200 bp and 400 bp corresponding to mono- and di-nucleosome footprints (Figure 2D, Figure 2F; Supplementary Fig. 2A). This organization remained stable throughout RS stages, indicating an intact nucleosome organization during this phase of development. However, as cells progressed to Early ES, the nucleosome-associated peaks were lost, and wider subnucleosomal peaks emerged (Figure 2D, 2E, Supplementary Figure 2A). These fragment sizes resemble destabilized nucleosome intermediates observed during active chromatin remodeling in other systems^42–44^. Curiously, these expanded sub-nucleosome peaks persisted through intermediate elongating spermatids (Int ES) but were lost in Late ES (Figure 2F). Concomitant with this loss, we observed a reemergence of mono-nucleosome-sized fragments in Late ES, suggesting re-stabilization of chromatin and selective retention of histones in a very small fraction of the mature sperm genome (Fig. 2D, Supplementary Fig. 2A).

To determine whether the loss of the nucleosome footprint in the early and Int ES was a biological state or a technical artifact, we leveraged the *Drosophila* spike-in data, which shows a well-defined nucleosome footprint across all samples (Figure 2B; Supplementary Figure 2B). Furthermore, when overlaying insert size distributions from all *Drosophila* spike-in datasets, there was no broadening or distortion of nucleosome peaks (Figure 2F), confirming that the loss of nucleosome footprint in ES isn’t due to excessive enzymatic digestion in some samples but a true biological chromatin state.

Having established this, we next asked whether it reflects a globally hyper-accessible chromatin state, perhaps coinciding with the previously described H4 hyperacetylated chromatin state known to loosen chromatin and facilitate histone-to-protamine exchange^45^. To estimate absolute accessibility, we normalized spermatid ATAC-seq signal to that of the *Drosophila* spike-in, which revealed that early and Int ES had markedly increased chromatin accessibility relative to other stages (Figure 2G). Notably, late RS displayed a similarly elevated accessibility relative to spike-in despite retaining canonical nucleosome organization, identifying a hyper-accessible yet nucleosome-preserved intermediate chromatin state (Figure 2G).

To determine how global changes in chromatin accessibility impact the organization of positioned nucleosomes, we generated insert size-resolved V-plots centered on annotated transcription start sites (TSS, ±200 bp), where assignment of genes to transcribed/active and non-transcribed/inactive states was based on previously published PRO-seq data from pooled round spermatids^46^. In RS (early through late), V-plots revealed a canonical promoter architecture characterized by well-positioned +1 and −1 nucleosomes flanking active TSSs (Figure 2H). As cells transitioned from late RS to the ES stage (Stage VIII), we first observed an attenuation of the di-nucleosome signal (earliest ES), followed by a complete loss of positioned nucleosomes (early & Int ES) (Figure 2H). Interestingly, a small fraction of positioned +1 nucleosomes re-emerges in Late ES, a trend that mirrors our genome-wide fragment length analysis (Figure 2D). In contrast, inactive gene promoters in RS lacked a defined nucleosome-free region (NFR) or structured promoters but had a diffuse nucleosome distribution pattern across the promoters, consistent with a transcriptionally silent architecture (Supplementary Figure 2C). Curiously, this disappearance of nucleosome-protected fragments was not restricted to active genes; as both transcribed and inactive promoters displayed a loss of a nucleosome footprint in the RS-to-ES transition, which likely reflects global hyper accessibility and destabilization of the chromatin landscape (Figure 2D, 2H).

Together, our time-resolved ATAC-seq analysis reveals multiple distinct states: a nucleosome-stable state in early and intermediate RS; a transient, hyper-accessible but nucleosome-stable state in late RS and the earliest ES stages; and a hyper-accessible and nucleosome-depleted phase during early-to-intermediate ES. In Late ES, a small but reproducible mono-nucleosome-sized fragment population re-emerges, consistent with partial chromatin re-stabilization and selective histone retention rather than de novo nucleosome assembly (**see below H3K27me3**).

### The spatiotemporal landscape of chromatin remodeling reveals asynchronous compaction of the spermatid genome.

**Figure 3:**
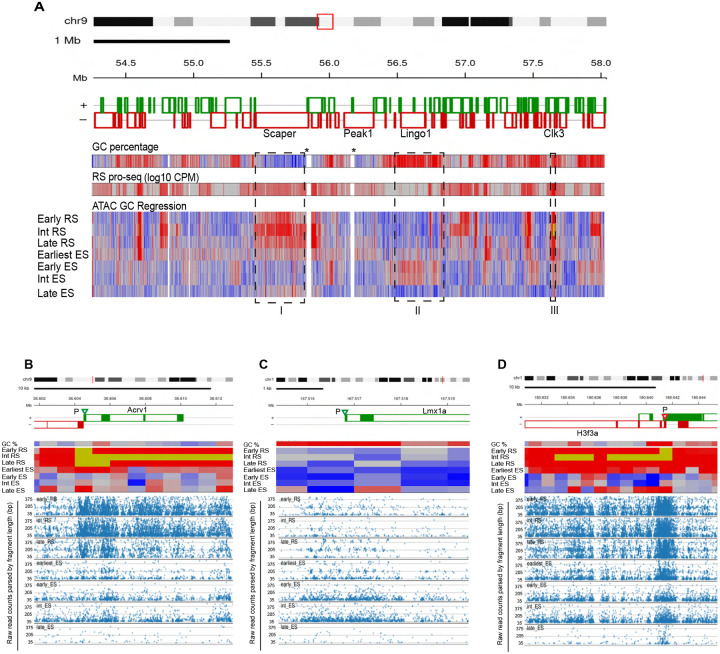
Genome-wide chromatin profiling reveals programmed, locus-specific remodeling during histone-to-protamine exchange. **(A)** Corrected ATAC-seq accessibility across a 4 Mb region of chromosome 9 for all seven spermatid stages (Early RS through Late ES). Tracks from top to bottom: GC content (red, GC higher than the grey genome median; blue, GC lower than median); PRO-seq signal from round spermatids (log_10_ CPM; red, transcribed)^30^; GC-residual ATAC-seq Z-scores per stage (red, relatively more accessible than the grey regression median, i.e., Z>0; blue, relatively less accessible, Z<0). Dashed boxes highlight three representative classes of locus behavior: progressive loss of accessibility in elongating spermatids (I); gain of accessibility in elongating spermatids (II); and stable accessibility throughout spermiogenesis (III). Asterisk (*) indicates blacklisted regions containing repetitive elements in the genome. **(B-D)** GC-residual ATAC-seq Z-scores (upper panels like (A); yellow, saturated high Z) and genomic V-plots across all seven stages (lower panels), for three loci exemplifying distinct accessibility trajectories. Each dot is a single mapped insert. Horizontal lines are at 35bp (minimum allowed fragment size), 205 bp (mono-nucleosome peak center), and 375 bp (di-nucleosome peak center). **(B)** *Acrv1* locus with high accessibility in round spermatids that is progressively lost during elongation. **(C)** *Lmx1a* locus with peak accessibility during the Late RS-to-Earliest ES transition. **(D)** *H3f3a* locus that maintains accessibility throughout spermiogenesis, with nucleosome occupancy selectively preserved at the promoter in Late ES.

To determine whether chromatin compaction in maturing spermatids follows a programmatic process, we analyzed chromatin accessibility changes within and across developmental stages at lower resolution. Because ATAC-seq can be influenced by GC content, and because chromatin composition changes markedly during spermatid maturation, we first examined GC-dependent biases across samples as this can confound comparisons across spermiogenesis (see **Methods**). When examining the ATAC-seq signal as a function of GC content across 1-kb genomic bins, we observed a progressive increase in GC-read count correlation from Early ES to Int ES (Supplemental Figure 2D). This pattern likely reflects the unusually accessible chromatin state at these stages, where increased enzyme accessibility amplifies intrinsic sequence preferences, leading to the exaggerated GC bias. To prevent these stage-specific biases from dominating inter-sample differences, ATAC-seq signal in 1kb bins were regressed as a function of GC content and converted to GC-normalized residual Z-scores. This normalization minimizes sequence-driven effects and facilitates direct comparison of relative chromatin accessibility across stages. A representative GC-normalized ~1 Mb region on chromosome 9 encompasses multiple genes and distinct patterns (Figure 3A; high Z-scores in red denote more accessible spans, while lower Z-scores in blue denote less accessible spans). Even within this small genomic interval, chromatin accessibility changes vary across loci and developmental stages. For example, Region I overlaps with the *Scaper* locus, which is transcribed in RS (Pro-seq track; red) and shows high accessibility early but progressively diminishes as spermatids elongate. In contrast, Region II, encompassing the non-transcribed gene *Lingo1*, is relatively inaccessible in round spermatids but transiently gains accessibility during early and Int-ES stages, when germ cells are transcriptionally quiescent, and returns to an inaccessible state in Late ES. Region III overlapping gene *Clk3* is highly transcribed in RS and remains accessible across all stages of spermatid development, including Late ES. Together, these representative patterns recur broadly across the genome and demonstrate that chromatin compaction during spermiogenesis proceeds in a region-specific and developmentally ordered manner rather than through uniform genome-wide silencing.

To examine local chromatin changes during spermiogenesis at higher resolution, we next generated genome-wide V-plots (Figure 3B–D; see **Methods**) to provide a read-level view of nucleosome composition or its disassembly intermediates across spermatid maturation at all loci. At the *Acrv1* locus, a highly transcribed acrosomal protein gene (Supplemental Figure 3A)^15^, V-plots revealed high accessibility in early stages (Early, In, Late RS; yellow heatmap is saturated high accessibility) followed by progressive loss of accessibility during elongation, consistent with transcription-dependent chromatin compaction (Figure 3B). However, the temporal ordering of *Acrv1* remodeling is graded. Nucleosome-sized fragments are first lost from the gene body and are only later lost from the promoter. In contrast, *Lmx1a*, a neuronal gene not transcribed in the male germline (Supplemental Figure 3B), follows a distinct chromatin remodeling trajectory (Figure 3C). Accessibility over the promoter region is diffuse and weakly structured in early and intermediate RS but becomes more pronounced and punctuated in late RS and earliest ES. By early and intermediate ES, the well-defined mono-nucleosome-sized fragments are lost, indicating a delayed, transcription-independent remodeling process (Figure 3C). At the ubiquitously expressed histone H3.3 gene, chromatin accessibility is maintained up to the Late ES stage, at which time gene body coverage is lost but promoter-centered nucleosome fragments are retained (Figure 3D; Supplementary Figure 3C). Finally, to confirm that the observed locus-specific patterns were not influenced by global differences in accessibility or sequencing depth across stages, we normalized insert-size counts to whole-genome signal within each developmental stage, which preserved all qualitative patterns (Supplementary Figure 3D; note samples sequenced to comparable depth). These examples show how chromatin remodeling during spermiogenesis proceeds through multiple, locus-specific patterns of chromatin accessibility gains and losses, reflecting both transcription-dependent and transcription-independent mechanisms. The switch of highest accessibility from region to region across spermiogenesis argues for a genome-scale, programmatic reorganization of chromatin rather than a uniform or stochastic compaction process.

### Peak-level chromatin dynamics support a continuous and programmed genome-wide remodeling during spermiogenesis.

**Figure 4:**
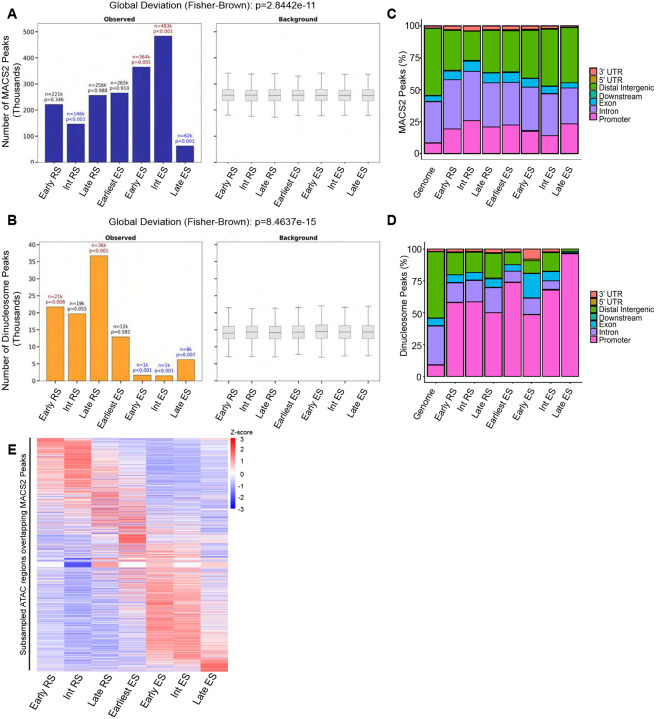
Genome-wide peak analysis confirms a programmed and continuous chromatin remodeling process, with selective retention of intact nucleosomes at promoters. **(A)** Bar plot of the number of accessible peaks identified by MACS2 at each spermatid stage (left), with observed peak counts annotated with Benjamini-Hochberg-adjusted p-values relative to a stage-matched null distribution (right). The global p-value (Fisher-Brown method, p=2.84×10^−11^) indicates that the overall distribution of peak counts deviates significantly from random expectation across spermiogenesis. **(B)** As in (A), for peaks identified by the dinucleosome-specific peak-calling pipeline (global Fisher-Brown p=8.46×10^−15^). **(C)** Genomic distribution of MACS2-called peaks across spermatid stages, expressed as proportion of peaks overlapping annotated genomic features. **(D)** Genomic distribution of dinucleosome-defined peaks across spermatid stages, as in (C). Both peak calling methods show enrichment at promoters relative to the genome average. **(E)** Heatmap of GC-regression-corrected ATAC-seq Z-scores at subsampled MACS2-defined peaks called in at least one stage. Each row is a subsampled peak overlap region ordered by the centroid of the stage-specific signals.

To further understand the unique properties of ATAC-seq read re-distribution during spermiogenesis, we next performed peak calling using two independent methods: (i) MACS2^47^ to identify regions of enriched accessibility above the genomic background (Figure 4A), and (ii) a dinucleosome-specific peak-calling algorithm that locates significantly accessible loci with multiple, well-positioned nucleosomes (Figure 4B, **see Methods**). The number of peaks identified by either method deviated significantly from random expectation (Figure 4A, global p=2.84e-11; Figure 4B, global p=8.46e-15). As expected, MACS2 identified more peaks, with the dinucleosome-defined peaks being entirely nested within the MACS2 set in every stage (Supplemental Figure 4A). Thus, while many regions are accessible, only a subset in each stage have the architectural stability of a multi-nucleosomal array. Despite the global chromatin remodeling and loss of nucleosome-sized fragments and positioned nucleosomes during spermatid elongation, the MACS2 peak number increased in early and intermediate elongating spermatids (Early ES: n=364k, p<0.001; Int ES: n=483k, p<0.001) before decreasing greatly in late elongating spermatids (Late ES: n=62k, p<0.001; Figure 4A). In contrast, regions maintaining stable dinucleosomes decreased the most in the Early ES and Int ES stages (Figure 4B). This inverse relationship between MACS and the dinucleosome algorithm indicates on a genome-wide scale that while Early ES and Int ES cells have highly accessible genomes, this accessibility lacks a stereotypical nucleosomal signature (Figure 2D, 2E, 4A, 4B). Notably, both methods identified a significant number of accessible loci in late ES, with peaks from each enriched at promoters relative to the genome average, especially the dinucleosome-enriched peaks (Figure 4C, D). When comparing dinucleosome accessible regions between RS and Late ES, we find that 6118 out of 6219 (98.4%) Late ES peaks overlapped a peak location seen in round spermatids, indicating that they do not represent acquired chromatin pattern during spermatid maturation but are a subset of early RS peaks that reemerge once the period of extreme accessibility resolves. Although round spermatid contamination can never be 100% ascertained, several lines of evidence argue against this interpretation: 1) we monitored purity of cells after FACs sorting, we pre-treated samples with DNase during sample preparation (reducing the possible of cell free chromatin), 3) when comparing global differences in chromatin accessibility patterns outside promoters using MACs peaks ~50% of loci overlap suggesting that these samples as whole differ from RS. Consistent with this, the B-compartment regions are only accessible in ES samples, not in RS (see below). Furthermore, gene ontology (GO) analysis for enriched promoters in all RS, early, and Int ES tended to enrich for the spermatogenesis program, DNA repair, and cilium formation (Supplementary Table 2), consistent with the expected developmental programming of these cells. In Late ES, GO recovered terms with well-established roles during embryonic development, including chromatin-remodeling, DNA repair, and developmental processes (Supplementary Table 2).

Finally, to characterize the temporal dynamics of ATAC-seq peaks, we either aggregated all MACS or all dinucleosome-defined peaks across stages and quantified their accessibility profiles over spermatid maturation. Consistent with the previous GC-normalized genome-wide patterns, these peaks display clear locus- and stage-dependent changes in chromatin accessibility. (Figure 4E; Supplementary Figure 4B). Principal component (Supplementary Figure 4C) and UMAP analysis (Supplementary Figure 4D) of peak-level accessibility reveal a continuous trajectory that parallels the temporal progression of spermiogenesis, with biological replicates clustered tightly and adjacent stages occupying neighboring positions along the trajectory. Together, these analyses indicate that the chromatin transition during spermiogenesis, even at peaks, occurs through a gradual, highly coordinated process, rather than a stochastic process.

### Chromatin remodeling in early spermatids is influenced by transcription, coincides with targeted histone eviction, but is not directed by H4 acetylation.

**Figure 5.**
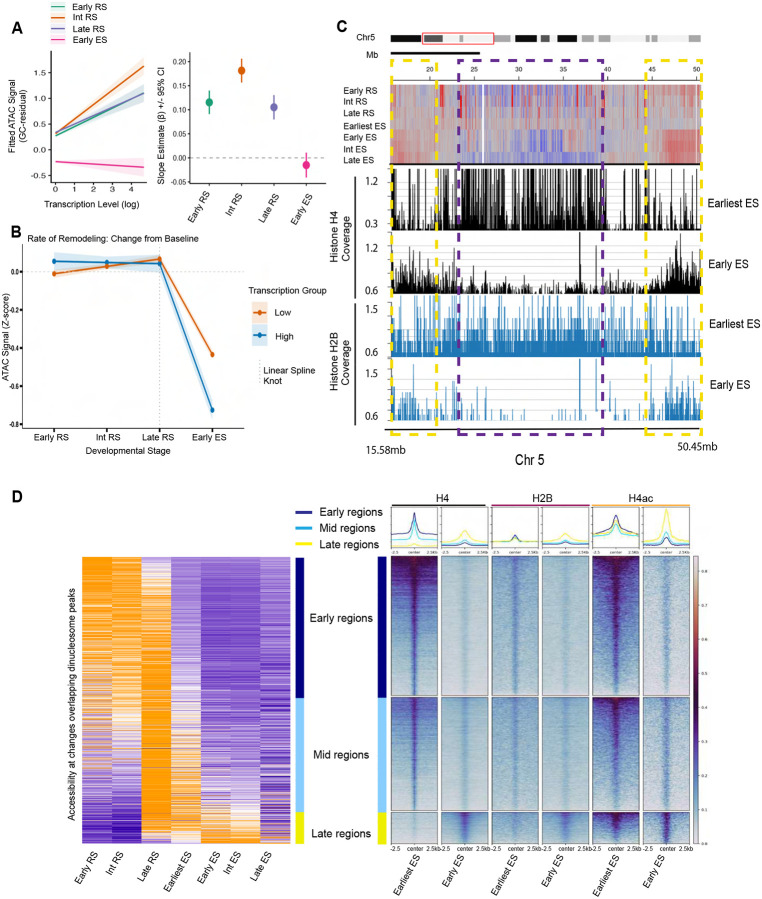
Chromatin accessibility loss during spermiogenesis is coupled to RS transcriptional activity and coincides with direct histone eviction. **(A)** Relationship between transcription level and chromatin accessibility across spermatid stages. Left: marginal predictions from a linear mixed-effects model showing fitted ATAC-seq signal (GC-corrected) as a function of transcription level (log_10_ CPM) at Early RS, Int RS, Late RS, and Early ES. Shaded ribbons denote 95% confidence intervals. The positive transcription-accessibility coupling present throughout RS stages (green, orange and purple) collapses to zero in Early ES (magenta). Right: stage-specific slopes (β) with 95% confidence intervals. **(B)** Model-estimating change in chromatin accessibility during spermatid development (GC-residual ATAC-seq Z-score) for genes in the top 5% of transcriptional activity (High; blue) and the 25–50th percentile (Low; orange) in Early RS. **(C)** GC-regression-corrected ATAC-seq Z-scores across a representative region of chromosome 5 (top), with CUT&Tag read coverage for histone H4 (black) and H2B (blue) at Earliest ES and Early ES shown below. Regions enclosed by purple dashed boxes or yellow dashed boxes exemplify differences in accessibility and histone eviction. **(D) (left)** Heatmap of GC-residual ATAC-seq Z-scores at dinucleosome-defined peaks. Each row is a subsampled peak overlap region ordered by the centroid of the stage-specific signals. Assigned groups based on dinucleosome peak calls reflect the temporal remodeling class: Early (accessibility lost first), Mid, and Late. **(right)** CUT& Tag meta-gene heatmaps for H4, H2B, and H4ac over Early, Mid, and Late remodeling regions at Earliest ES and Early ES.

To understand whether the accessibility changes observed between late round/earliest ES (spermatids in stages 7–8) and Early ES (spermatids in stage 9–12) are transcription-dependent, we integrated our GC-normalized ATAC-seq profiles with stage-matched scRNA-seq centroids^48^ and quantified the coupling between promoter accessibility and gene expression across spermatogenic stages. Across all round spermatid stages, promoter accessibility and transcription dynamics are tightly coupled (Figure 5A, Supplementary Figure 5A). In contrast, this coupling between chromatin accessibility and transcription was lost in Early ES (Figure 5A, Supplementary Figure 5A); consistent with the known transcriptional quiescence in elongating spermatids. Interestingly, genes in the top 5% of transcriptional activity in early RS not only show a steeper decline in accessibility during elongation than moderately transcribed genes (25–50th percentile), but by Early ES they become less accessible than regions that were previously lowly transcribed - a reversal that cannot be explained simply by their higher starting accessibility (Figure 5B). This suggests that highly transcribed genes are preferential substrates for chromatin remodeling during the RS-to-ES transition, and that prior transcriptional activity may license or direct early compaction.

Given the loss of transcription-accessibility coupling in ES, we next examined whether the loss of accessibility coincides with changes in chromatin composition, which would be consistent with PRM1 incorporation during very late rounds and early elongation. This interpretation is consistent with the observed onset of PRM1 incorporation during very late rounds and early elongating (Figure 1B). To test whether accessibility loss corresponds with histone eviction, we profiled histone H4 and H2B by CUT&Tag in late rounds/earliest elongating or early elongating spermatids and compared histone abundance at early-, mid-, and late remodeling loci defined from our ATAC-seq dataset. Regions that lose relative accessibility from late rounds/earliest ES to Early ES also lose both H2B and H4 signal as compared to adjacent regions (Figure 5C). This pattern is not specific to a representative locus on chromosome 5 but is seen at all early- and mid-remodeling loci (Figure 5D), suggesting that accessibility loss in Early ES is likely due to nucleosome eviction and transition to a less accessible epigenomic state. To determine what might guide this selective histone eviction, we examined histone H4 acetylation - a modification associated with chromatin loosening and proposed to facilitate histone-protamine exchange during spermatid remodeling across multiple species^45,49–51^. CUT&Tag profiling of H4ac in late RS/earliest ES shows that H4ac, like H4 and H2B, is broadly distributed across all groups, with comparable enrichment in regions regardless of whether regions remodel early or late (Figure 5D; right panel). In Early ES, H4ac is markedly reduced in early- and mid-remodeling regions but is selectively retained at late-remodeling loci. Thus, H4ac marks chromatin competent for eviction but does not specify the temporal order of eviction, implying that additional mechanisms dictate the spatiotemporal hierarchy of chromatin remodeling (Figure 5D).

### Large-scale programmatic closing of the spermatid genome is defined by A and B compartments.

**Figure 6:**
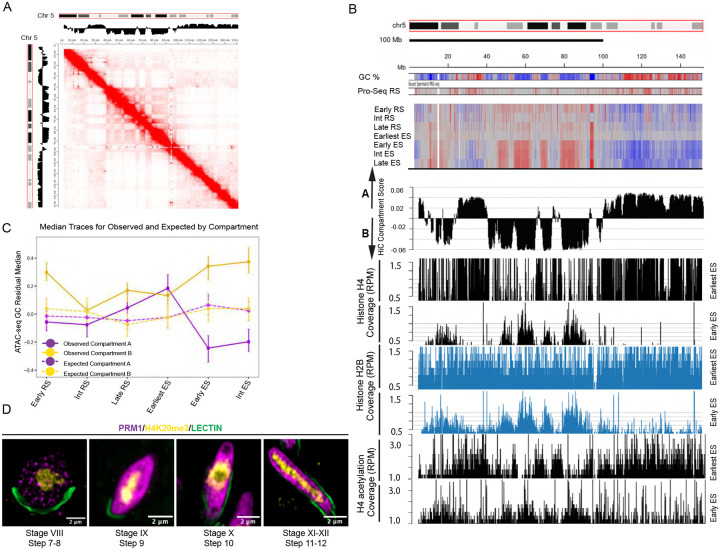
Chromatin compartment identity governs the spatial and temporal order of histone-to-protamine exchange. **(A)** Hi-C contact matrix of intrachromosomal interactions on chromosome 5 from round spermatids, with the corresponding A/B compartment track shown above and to the side (positive values, A compartment; negative values, B compartment). **(B)** Track view of chromosome 5 showing, from top to bottom: GC content per bin; PRO-seq signal from round spermatids; GC-regression-corrected ATAC-seq Z-scores across all seven spermatid stages; Hi-C compartment scores (A compartment, positive; B compartment, negative); CUT&Tag read coverage (reads per million, RPM) for histone H4 at Earliest ES and Early ES (black); CUT& Tag read coverage for histone H2B at Earliest ES and Early ES (blue); and CUT& Tag read coverage for H4 acetylation at Earliest ES and Early ES (black). Heat map coloring is the same as Fig. 3A. **(C)** Median GC-regression-corrected ATAC-seq Z-scores across spermatid stages for genomic regions in the A compartment (solid purple) and B compartment (solid gold), with expected values from a standard normal distribution shown as dashed lines. Error bars represent 95% confidence intervals estimated by bootstrap resampling. **(D)** Immunofluorescence of individual spermatids isolated from specific stages of the seminiferous tubule cycle: Roman numbers are representative of the seminiferous tubule cycle stages, and Arabic numerals indicate spermatid steps in the 12 stages. Germ cells were co-stained for PRM1 (magenta, via V5 tag) and H4K20me3 (yellow), with Lectin (green). Scale bar: 2 μm.

Although individual loci display stage-specific accessibility changes during spermiogenesis (Figure 3), chromatin changes appear to be coordinated over local contexts. To quantify this coordination, we performed a variogram analysis, which showed that variance increases with inter-peak distance up to a plateau of maximal inter-peak variance at ~100 kb (Supplementary Figure 6A), indicating that nearby regions tend to remodel together over a characteristic neighborhood-scale length. To test whether nuclear organization of chromatin may facilitate this coordination, we generated four Hi-C datasets from round spermatids (day 35 and day 39 post-synchronization), prior to the onset of histone-to-protamine exchange, to delineate round spermatid-specific A/B compartments. Biological replicates showed high concordance (HiCRep stratum-adjusted correlation coefficients of ~0.83–0.92; Supplementary Figure 6B), providing justification to combine and create a single high-resolution round-spermatid Hi-C dataset totaling ~2.3 billion alignable reads (**see Methods**). As expected, the resulting Hi-C contact maps revealed strong A/B compartmentalization and preferential self-interaction within compartments (Figure 6A). We confirmed that the defined compartments have the predicted genomic features, e.g., A-compartment regions are enriched for higher GC content (%GC track) and transcriptional output (PRO-seq track), whereas B-compartment regions displayed the opposite pattern (Figure 6B).

When comparing genome-wide accessibility dynamics across compartments, we observe a clear difference in remodeling behavior at a low-resolution scale: A-compartment regions progressively lose relative accessibility as cells transition from late round to elongating spermatids, whereas B-compartment regions retain higher accessibility well into later elongating spermatid (Figure 6B). This compartment-specific pattern is observed across multiple chromosomes (Supplementary Figure 6C). Consistent with the representative chromosomal snapshots, the median accessibility levels for A-compartment loci drops quickly between the earliest and early elongating spermatid stages, whereas B-compartment regions retain higher accessibility well into late spermatids (Figure 6C). Notably, these distinct patterns observed are different from the expected background distributions, where both A and B compartments have median accessibility of around zero across all stages often with their confidence intervals overlapping (see **Methods**). To validate that the A compartment regions are likely to remodel first in vivo, we co-stained PRM1 with the heterochromatin mark H4K20me3 and monitored PRM1 localization across successive stages of elongating spermatids using structured illumination microscopy (SIM) (Figure 6D). In stage VIII–IX spermatids, PRM1 appears as discrete puncta localized to euchromatic regions and is clearly excluded from the H4K20me3-rich chromocenter. By stage IX-X, PRM1 spreads more broadly throughout the nucleus while still avoiding the heterochromatic center, and by stages XI–XII, PRM1 uniformly fills the elongating nucleus yet remains depleted from the chromocenter. Taken together, the in vivo and molecular data indicate that the timing of early versus late chromatin remodeling is strongly aligned with - and likely constrained by - the underlying 3D genome architecture established in round spermatids.

Consistent with these accessibility dynamics across compartments, A-compartment regions that lose chromatin accessibility during the transition from late rounds/earliest ES to Early ES also show parallel losses in relative histone H2B and H4 occupancy (Figure 6B). Tracking histone H4ac across compartments and stages reveals similar patterns. At late rounds/earliest ES (stages 7–8 spermatids), kernel density plots of H4ac suggest that H4ac levels are higher in A than in B compartments (Supplementary Figure 7A). However, this enrichment in the A compartment appears to be driven by the greater number of H4ac peaks in A compared with B (Supplementary Figure 7A). Furthermore, we find that the GC-normalized ATAC-seq distributions for H4ac-enriched loci in both compartments are remarkably similar and overlapping (histograms and box plots); suggesting that although H4ac sites are equally accessible in both compartments, they are selectively lost from the A compartment as cells enter Early ES. Consequently, by Early ES, the remaining H4ac signal is disproportionately retained in B-compartment regions (density plot; histogram; Supplementary Figure 7A). This apparent “shift” of H4ac toward B compartments is likely due to compositional consequences of differential histone loss and early closure of the A compartment. Consistent with this observation, in Early ES, the H4ac peaks that persist show notable differences in accessibility: those that persist in B compartments remain significantly more accessible than those few retained in A, indicating that once A-compartment chromatin begins its programmed closure, the presence of residual H4ac is insufficient to maintain accessibility.

### A proportion of H3K27me3-modified nucleosomes are stably maintained during spermiogenesis.

A small proportion of nucleosomes is retained in mature sperm in a species-specific manner, with approximately 1–5% in mouse and 10–15% in human^14–16^. The large proportion of nucleosomes retained in mature sperm bear histone H3 lysine 27 trimethylation (H3K27me3), and include developmentally important loci, i.e., the Hox clusters, in both species^14–16,21^. Whether these retained nucleosomes reflect stable inheritance throughout the histone-to-protamine exchange process or are re-established at the end of spermiogenesis is unknown. To distinguish between these possibilities, we profiled H3K27me3-marked nucleosomes by CUT&Tag across Early RS, Late RS, Earliest ES, Early ES, and Int ES. Consistent with observations in mature sperm, we detected robust signal and enriched peaks at developmental promoters, including the *Hoxd* cluster; even when nucleosomes are destabilized (Supplementary Figure 7B; Supplementary Table 3). In contrast to H4-acetylated regions, which shift from A-to-B compartment in Early ES (Supplementary Figure 7A), H3K27me3-marked regions were maintained in the A compartment between spermatid transitions (Supplementary Figure 7C) and retained similar chromatin accessibility dynamics in both spermatid stages, unlike H4ac (Supplementary Figure 6A). To quantify the fraction of H3K27me3-marked loci stably maintained across spermiogenesis, we determined the proportion of peaks defined in Early RS that persisted through the ES stages. Approximately 60% of H3K27me3 peaks were retained (Supplementary Figure 7D), consistent with the possibility that these loci contribute to the retained nucleosome pool present in mature sperm. The removal of some H3K27me3-marked loci appears to be functionally important because mouse embryos generated by round spermatid injection (ROSI), which bypass the completion of histone-to-protamine exchange, exhibit ectopic retention of H3K4 and H3K27 methylation and show widespread misregulation of developmental genes, contributing to the low developmental competence of ROSI-derived embryos^52^. Complementary evidence from Xenopus has demonstrated that active demethylation of H3K4 and H3K27 in maturing spermatids is required for normal embryo viability^20^. Together, these findings suggest that H3K27me3 alone is insufficient to predict nucleosome inheritance in mature sperm; rather, the precise remodeling of both H3K4 and H3K27 methylation states during spermiogenesis is essential for establishing the epigenetic landscape required for embryonic development.

## Discussion:

**Figure 7:**
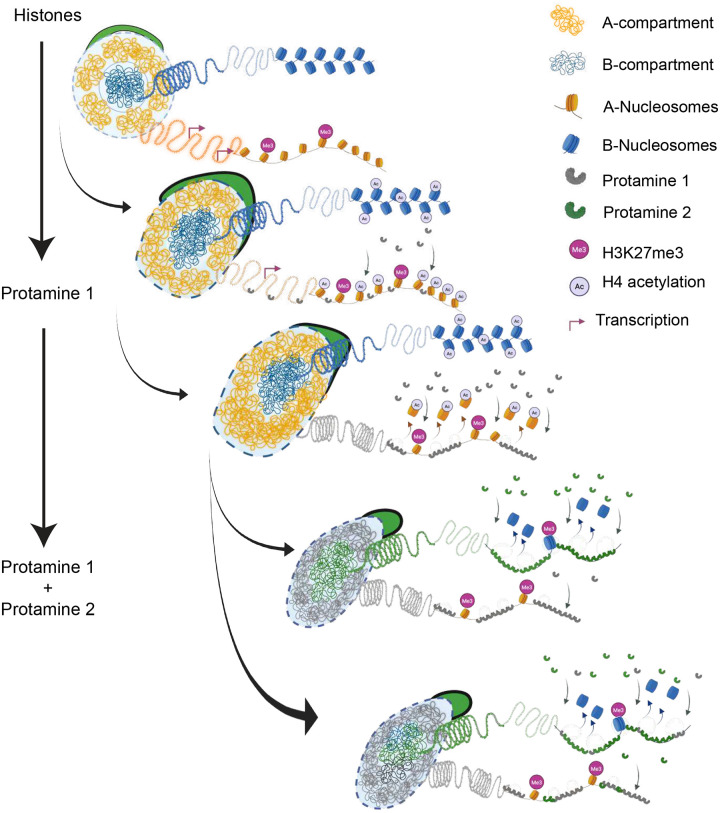
Model for compartment-encoded, programmatic histone-to-protamine exchange. This model illustrates a staged, compartment-resolved progression of chromatin remodeling during spermiogenesis. In late RS H4 hyperacetylation spreads globally, both in early and late remodeling loci, yet histone eviction and compaction initiate in the A-compartment chromatin, while B-compartment chromatin regions remain accessible until Late ES. Notably, A-compartment compaction coincides with PRM1 incorporation, whereas PRM2 appears late in spermatid development after TNPs, likely initiating a second, spatially distinct wave of compaction, potentially restricted to the B-compartment. The closure of the B compartment is predicted to be mediated either entirely by PRM2 or a combination of PRM1 and PRM2. Together, these findings define a programmed remodeling cascade in which the pre-existing three-dimensional nuclear architecture of the round spermatid nucleus dictates the order, timing, and spatial logic of histone eviction and protamine deposition.

Protamines are the primary chromatin packaging proteins in sperm, yet, unlike histones, their deposition onto the paternal genome has been presumed to occur through nonspecific electrostatic interactions.^1^ Here we revisit this assumption and ask whether protamine-mediated genome packaging in sperm is random or programmed. Using endogenously tagged *Prm1* and *Prm2* mouse models, we demonstrate that the histone-to-protamine transition proceeds through a direct histone-to-PRM1 exchange, whereas TNPs precede only PRM2 incorporation. These findings demonstrate that PRM1 and PRM2 incorporation are temporally and, likely, mechanistically uncoupled, which revises the earlier model for Histone-to-Protamine exchange. To define the underlying molecular logic underlying the genome-wide chromatin remodeling, we mapped chromatin accessibility at high temporal resolution across spermatid maturation, and show that chromatin compaction is spatiotemporally asynchronous, proceeds through both transcription-dependent and transcription-independent mechanisms, and is facilitated but not directed by H4 hyperacetylation. Most strikingly, the order of compaction is strongly associated with the pre-existing 3D compartment architecture of the round spermatid genome. Together, these findings further underscore a programmatic nature and a compartment-correlated blueprint for establishing the paternal epigenome. (Figure 7)

The differential timing of PRM1 and PRM2 nuclear localization has been previously documented by antibody staining^7^ but has never been mechanistically interrogated or integrated into the canonical remodeling framework. Our endogenously tagged protamine models, combined with combinatorial staining, provide greater resolution of this revised histone-to-protamine exchange sequence. Genetic evidence supporting a direct histone-protamine1 exchange comes from *Tnp1/2* double-knockout mice, which showed normal PRM1 incorporation timing and levels, but also marked reductions in PRM2 incorporation, and, in some spermatids, a complete loss of PRM2^53^. This observed phenotypic difference in PRM1 and PRM2 regulation in these double TNP mutants can now be explained by the differential timing of PRM loading we observe and by the TNP-independent deposition of PRM1. This establishes PRM1 and PRM2 incorporation as mechanistically distinct events rather than steps in a single process. Further, this surprising, programmed uncoupling of PRM1 and PRM2 raises questions: do PRM1 and PRM2 occupy distinct genomic loci, and if so, what mechanisms direct each protamine to its target loci?

In early elongating spermatids, we find that PRM1 is recruited to H4-hyperacetylated chromatin. This early incorporation of PRM1 is likely mediated by BRDT, as deletions of the first bromodomain (BD1) have been shown to prevent the incorporation of PRM1^54–56^. Specifically, it has been proposed that nucleosomes bearing H4K5/K8 acetylation are recognized by BRDT, removed, and targeted for degradation by the PA200-containing proteasome^56^. Whether the BD1 domain of BRDT is similarly required for PRM2 incorporation remains to be determined; however, other histone modifications, such as H2BK12 crotonylation, have been implicated in TNP and PRM2 deposition in elongating spermatids^57^.

A key unresolved question is what regulates the stage-specific remodeling patterns we observe during spermiogenesis, since H4 hyperacetylation is broadly distributed across the genome in early and late remodeling regions as soon as it is established. Elegant work by Saadi Khochbin’s lab may provide insights into this sequence of remodeling events: H4K5/K8 butyrylation frequently co-occurs with H4 acetylation, and both exist independently at a subset of loc^56^. Early biochemical experiments show that the combination of H4ac and H4 butyrylation inhibits BRDT binding^56^. This suggests a combinatorial code in which acetylation, butyrylation, and crotonylation likely function as molecular timers that determine where and when structural exchange occurs, which inturn likely bias PRM1 vs. PRM2 incorporation.

By leveraging ATAC-seq insert size distributions, we provide insights into the underlying chromatin states during the histone-to-protamine transition. In early and Int-ES, we observed a marked loss of the canonical ~147-bp nucleosome footprint, coincident with H4 hyperacetylation and the onset of PRM1 incorporation. Concurrently, the insert size distribution shifts towards a prominent ~70–90 bp sub-nucleosome species, which is substantially wider than the TF-protected fragments or nucleosome-free region (NFR) in round spermatids. This subnucleosomal intermediate likely reflects destabilized nucleosomal states^43^ or disassembly intermediates, such as hexasomes or tetrasomes, generated by the loss of one or both H2A-H2B dimer^58–60^. However, we also cannot exclude the possibility that some fraction of this signal reflects protamine-bound DNA fragments. Distinguishing between these possibilities will require orthogonal approaches, such as gentle MNase-based footprinting combined with nucleosome mass spectrometry^61^ or direct protamine mapping using single-molecule sequencingapproaches^62^. Both are extremely challenging due to cell number requirements^61^ or technical reasons, due to the way protamine coats DNA^36^. However, the remodeling intermediate interpretation is nonetheless supported by evidence from other systems. In *Drosophila*, H2A-H2B removal precedes H3-H4 loss, generating a temporal series of disassembly intermediates during the transition to sperm nuclear binding proteins (SNBP)^59^. In *pal* mutants, H2A-H2B are removed normally, but H3-H4 tetramers are retained, yet this doesn’t impair SNBP loading, demonstrating that H3-H4 tetramers can coexist with SNBP-packaged chromatin^59^. A similar finding is observed in *Xenopus*, where H2A-H2B dimers are lost during spermatid maturation, but H3-H4 tetramers are selectively retained^60^. Whether these particles represent bona fide hexasome or tetrasome intermediates, protamine-bound DNA of similar fragment size, or destabilized full nucleosomes with impaired DNA wrapping - as proposed for remodeler-engaged nucleosomes in other systems - remains to be determined by systematic proteomic and single-molecule analyses.

A striking feature of early PRM1 incorporation is its punctate, euchromatin-restricted nuclear pattern, raising questions about how protamines first nucleate into these genomic regions. Our cross-correlations between ATAC-seq and scRNA-seq expression data across spermatid stages allow us to propose several potential models. In round spermatids, chromatin accessibility is tightly coupled to transcriptional activity, as expected. However, in Early ES, the most highly transcribed regions undergo a precipitous loss of relative accessibility - a transition that coincides precisely with the onset of punctate PRM1 incorporation. This could reflect a passive consequence of global chromatin reorganization, in which the rest of the genome becomes more accessible as elongation proceeds. However, we favor a more direct interpretation: that PRM1 is preferentially loaded into highly transcribed regions by virtue of their elevated accessibility because of nucleosome turnover or, more intriguingly, through transcription-coupled DNA strand breaks that create entry points for protamine intercalation. The latter is a more exciting possibility given that it has long been observed that post-meiotic round spermatids are known to have pervasive transcription^63,64^, yet its functional significance is unknown. Although prior assumptions suggest that this pervasive transcription is a ‘leaky’ byproduct of chromatin remodeling during spermiogenesis; we propose that this transcriptional activity is not incidental but is coupled to the initiation of histone-to-protamine exchange to establish a transcription-coupled chromatin remodeling pathway. Importantly, this mechanism accounts only for the remodeling of active genomic domains. The rapid and global decompaction of the B-compartment in Int- and late-ES suggests a parallel, distinct regulatory mechanism governing decompaction and re-compaction of heterochromatin. We speculate that this transition is driven by the programmed loss of heterochromatin-specific architectural factors, which, upon their removal, render these previously sequestered regions accessible to the nascent PRM 2 machinery. Thus, the genome-wide transition to a protamine-packaged state is a multi-step process: one governed by transcription-coupled remodeling in the A-compartment, and a second, independent pathway - potentially driven by the dissociation of repressive complexes - that triggers de-compaction across the B-compartment.

Whether protamines possess intrinsic DNA-sequence preferences or are guided to nucleation sites by dedicated chaperones remains unresolved. Protamines, like other basic proteins or zinc fingers, may have a modular ‘recognition code^65,66,67^. Indeed, simulation studies using arginine-rich short cationic peptides with varying numbers and spacing of consecutive arginine residues bias interactions toward GC-rich major-groove or AT-rich minor-groove in the double helix.^68^ Furthermore, in the real protein context in vivo, the protamine protein binding preference is also likely modulated by post-translational modifications that we and others have identified^69–71^. Once these proteins bind DNA, they appear to bind cooperatively^70,72,73^ – beginning with a nucleation event that spreads outward - a behavior supported by our earlier in vitro biophysical assessments on DNA curtains^70^ and by our imaging data here. This cooperative spreading is consistent with a bind-and-bend mechanism, in which initial protamine binding induces a local DNA conformational change that increases the probability of subsequent protamine binding and loop expansion.^72^ Consistent with this, we observe dense clusters of PRM1 in early spermatids, which become more diffuse in later stages. However, stably retained nucleosomes, particularly those marked by H3K27me3, could act as barriers to protamine domains, a finding consistent with previous models.^36^

Although locus-specific genomic/biochemical cues clearly shape where and when protamines load, our data reveal a broader organizational principle that correlates with and likely reflects the re-established A/B compartment structure of the round spermatid genome. This compartment structure, which is reinstated following its dissolution during meiosis, serves as a powerful predictive blueprint for how chromatin is remodeled: i.e., A-compartment regions compacting earlier than B-compartment regions, creating a spatially coherent, genome-wide progression that can’t be driven by local histone modification alone. This re-established chromatin compartment identity in rounds is not a transient feature of spermiogenesis; it is maintained/strengthened in mature sperm and directly passed on to the paternal pronucleus of the one-cell zygote.^74,75–77^ These observations suggest that sperm can deliver more than DNA methylation and a small fraction of retained histones, but rather provide compartment-level information to the zygotes that is encoded by PRM1 vs PRM2. In line with this observation, it has been shown in mouse embryos that the paternal pronucleus has distinct chromatin compartments, whereas maternal pronuclei do not.^76^ Furthermore, the emerging compartment landscape, starting with the two-cell embryo, appears to follow the compartmental patterns observed in mature sperm, suggesting the exciting possibility of a paternal epigenome-guided chromatin organization in the embryo.^75^ Furthermore, it has also been shown that A and B compartments precede, guide zygotic transcription, and instruct replication timing in early embryos.^78^ Together, these observations suggest that higher-order chromatin organization established in spermiogenesis could have implications in early embryo development.

## Supplementary Material

Supplementary Files

This is a list of supplementary files associated with this preprint. Click to download.

- SupplementaryTable1.xlsx
- SupplementaryTable2.xlsx
- SupplementaryTable3.xlsx
- CombinedSupplementaryMaterials7826.pdf
